# Differences in the pharmacokinetics of currently approved antimalarial drugs in uncomplicated malaria patients compared to healthy subjects

**DOI:** 10.1186/1475-2875-11-S1-P118

**Published:** 2012-10-15

**Authors:** Jay Prakash Jain, Suresh B Lakshminarayana, Gilbert Lefèvre, Rama Sivasubramanian, Francesca Blasco, Gangadhar Sunkara

**Affiliations:** 1 Novartis Healthcare Private Limited, Hyderabad, India; 2 Novartis Institute for Tropical Diseases Pte Ltd, Singapore Country Singapore; 3 Novartis Pharma AG, Basel, Switzerland; 4 Novartis Institute for Biomedical Research, East Hanover, NJ, USA

## 

For an effective antimalarial therapy, maintenance of antimalarial drug concentrations well above minimum parasiticidal concentration for certain time duration is required in the target compartments (eg., blood, liver). On the other hand, malaria parasite infection may affect few physiological functions (eg., hepatic metabolism, protein binding) that may affect the pharmacokinetics and tissue disposition of antimalarial drugs. The altered PK in turn can affect the concentrations of the drugs and consequently their efficacy. Therefore, this work was undertaken to understand whether there is any difference in the pharmacokinetics (PK) of currently approved/available antimalarial drugs between malaria patients and healthy subjects.

The pharmacokinetic data of approved drugs was obtained from various public resources and the data was compiled for absorption, distribution, metabolism and elimination (ADME) properties. These ADME properties were further analyzed against the information available on reported physiological changes (e.g. reduced hepatic blood flow in patients, changes in CYP enzyme levels, etc.) to understand the possible contributing factors for potential alterations in the pharmacokinetics of drugs in patients. Pre-clinical information available for these drugs were also retrieved and used for the present investigation.

The results indicated that there was a significant alteration in the pharmacokinetic properties of most of the currently available/ approved antimalarial drugs in malarial patients compared to healthy subjects. The correlation analysis indicated that physiological changes such as hepatic blood flow, CYP enzyme expression/activity and protein binding may be the potential reasons for the observed differences. This analysis could be useful to envisage changes in the PK properties of the drugs based on their ADME properties and further aid in the development of future antimalarial drugs.

